# Pramipexole enhances levodopa's therapeutic efficacy in Parkinson's disease: Role of glutaredoxin-1 (GRX1), peroxiredoxin3 (PRX3), thioredoxin (TRX), 8-hydroxy-2 -deoxyguanosine (8-OHDG), and neurosteroid dehydroepiandrosterone sulfate (DHEA-S)

**DOI:** 10.5937/jomb0-59700

**Published:** 2026-03-17

**Authors:** Yu Shu, Jinjun Qian

**Affiliations:** 1 The Fourth People's Hospital of Zhenjiang, Department of Neurology, Zhenjiang, China

**Keywords:** Parkinson's disease, Levodopa, pramipexole, oxidative stress, glutaredoxin-1, peroxiredoxin-3, thioredoxin, 8-OHdG, neurosteroids, cognition, motor function, UPDRS, MMSE, MOCA, Parkinsonova bolest, levodopa, pramipeksol, oksidativni stres, glutation-reduktaza-1, peroksiredoksin-3, tioredoksin, 8-OHdG, neurosteroidi, kognicija, motorna funkcija, UPDRS, MMSE, MoCA

## Abstract

**Background:**

Parkinson's disease (PD) is characterised byprogressive neurodegeneration and dopamine deficiency.Levodopa remains a cornerstone treatment, but its longterm use is associated with motor complications andreduced efficacy. This study investigates the clinical benefits of combining pramipexole with Levodopa in PDpatients, focusing on improvements in oxidative stress,cognitive function, and motor control. Additionally, thestudy explores the role of novel biomarkers, includingglutaredoxin-1 (Grx1), peroxiredoxin-3 (Prx3), thioredoxin(Trx), 8-hydroxy-2'-deoxyguanosine (8-OHdG), and neurosteroid dehydroepiandrosterone sulfate (DHEA-S), inevaluating oxidative stress and neuroprotection.

**Methods:**

A total of 92 PD patients were enrolled andassigned to either a levodopa monotherapy group (n=46)or a pramipexole-levodopa combination group (n=46).Clinical efficacy was assessed using the Unified Parkinson'sDisease Rating Scale (UPDRS). Cognitive function wasevaluated using the Mini-Mental State Examination(MMSE) and the Montreal Cognitive Assessment (MoCA).Oxidative stress markers (SOD, GSH, GSH-PX, CAT, alongwith Grx1, Prx3, Trx, and 8-OHdG) were measured inserum samples. Additionally, DHEA-S was analysed as aneurosteroid biomarker to assess its potential role in cognitive and motor function improvements. Quality of life(QOL) was evaluated using the PDQ-39 questionnaire.

**Results:**

The combination therapy group exhibited a significantly higher effective rate (93.48% ) compared to the levodopa group (78.26% ) (P&lt; 0.05). UPDRS scores were significantly lower in the combination group at 6- and12-week post-treatment (P &lt; 0 .0 5 ). The combinationgroup also showed a significantly lower incidence ofadverse drug reactions (6.52% vs. 23.91% , P&lt; 0.05). After3 months, the combination group displayed significantlyhigher levels of SOD, GSH, GSH-PX, CAT, Grx1, Prx3, andTrx, while 8-OHdG levels were significantly reduced, indicating enhanced neuroprotection and reduced oxidativestress. DHEA-S levels were also elevated, correlating withimproved MMSE and M oCA scores (P &lt; 0 .0 5 ). Theobserved DHEA-S elevation in the combination group maybe due to pramipexole's dopaminergic modulation ofhypothalamic-pituitary-adrenal axis activity, potentiallyenhancing adrenal steroidogenesis. Alternatively, improvements in motor and cognitive function may reduce chronicstress, indirectly elevating DHEA-S. It suggests a neurosteroid-mediated cognitive benefit. QOL was significantlybetter in the combination group after 3 months of intervention (P&lt; 0.05).

**Conclusions:**

Pramipexole combined with Levodopa significantly improves clinical outcomes, reduces adverse effects,enhances cognitive function, and alleviates oxidative stressin PD patients. The inclusion of novel biomarkers such asGrx1, Prx3, Trx, 8-OHdG, and DHEA-S provides deeperinsight into the molecular mechanisms underlying thesetherapeutic effects. This combination therapy represents avaluable strategy for improving PD management and warrants wider clinical application.

## Abstract (sr)

**Uvod:**

Parkinsonova bolest (PB) se karakteriše progresivnom neurodegeneracijom i nedostatkom dopamina. Levodopa ostaje osnovni lek u terapiji, ali njena dugotrajnaprimena je povezana sa motornim komplikacijama i smanjenom efikasnošću. Ova studija ispituje kliničke koristikombinovanja pramipeksola sa levodopom kod pacijenatasa PB, sa fokusom na poboljšanje oksidativnog stresa, kognitivne funkcije i kontrole pokreta. Takođe se istražujeuloga novih biomarkera, uključujući glutation-reduktazu-1(Grx1), peroksiredoksin-3 (Prx3), tioredoksin (Trx), 8-hidroksi-2'-dezoksiguanozin (8-OHdG) i neurosteroiddehidroepiandrosteron-sulfat (DHEA-S), u proceni oksidativnog stresa i neuroprotekcije.

**Metode:**

U studiju je uključeno ukupno 92 pacijenta sa PB,koji su raspoređeni u grupu sa monoterapijom levodopom(n=46) ili u grupu sa kombinacijom pramipeksol-levodopa(n= 46). Klinička efikasnost je procenjivana pomoću Uni -fikovane skale za procenu Parkinsonove bolesti (UPDRS).Kognitivna funkcija je procenjivana pomoću Mini-mentalnog testa stanja (MMSE) i Montrealske skale kognitivneprocene (MoCA). Markeri oksidativnog stresa (SOD, GSH,GSH-Px, CAT, zajedno sa Grx1, Prx3, Trx i 8-OHdG) mereni su u uzorcima seruma. DHEA-S je analiziran kao neurosteroidni biomarker radi procene njegove moguće ulogeu poboljšanju kognitivne i motorne funkcije. Kvalitet života(QOL) je procenjivan korišćenjem upitnika PDQ-39.

**Rezultati:**

Grupa sa kombinovanom terapijom je pokazalaznačajno visu stopu efikasnosti (93,48% ) u poređenju sagrupom koja je primala samo levodopu (78,26% )(P&lt; 0,05). UPDRS skorovi bili su značajno niži u kombinovanoj grupi nakon 6 i 12 nedelja terapije (P&lt; 0,05).Takođe, u kombinovanoj grupi je zabeležena značajno nižaučestalost neželjenih reakcija na lek (6,52% naspram23,91% , P&lt; 0,05). Nakon 3 meseca, kombinovana grupapokazala je značajno vise nivoe SOD, GSH, GSH-Px, CAT,Grx1, Prx3 i Trx, dok su nivoi 8-OHdG bili značajno smanjeni, sto ukazuje na poboljsanu neuroprotekciju i smanjenoksidativni stres. Nivoi DHEA-S su takođe bili povecani ikorelirali su sa poboljsanim MMSE i MoCA skorovima(P&lt; 0,05). Povecanje DHEA-S u kombinovanoj grupi možeda bude posledica dopaminergičke modulacije aktivnostihipotalamusno-hipofizno-adrenalne ose prouzrokovanepramipeksolom, čime se potencijalno podstiče adrenalnasteroidogeneza. Alternativno, poboljsanja motorne i kognitivne funkcije mogu smanjiti hronični stres, sto indirektnopodiže nivoe DHEA-S. Ovo ukazuje na kognitivnu koristposredovanu neurosteroidima. Kvalitet života je bio značajno bolji u kombinovanoj grupi nakon tromesečne intervencije (P&lt; 0,05).

**Zaključak:**

Kombinovana terapija pramipeksol-levodopaznačajno poboljsava kliničke ishode, smanjuje neželjeneefekte, unapređuje kognitivne funkcije i ublažava oksidativni stres kod pacijenata sa PB. Uključivanje novih biomarkera (Grx1, Prx3, Trx, 8-OHdG i DHEA-S) pruža dubljiuvid u molekularne mehanizme ovih terapijskih efekata.Ova terapijska strategija predstavlja značajan pristup ulečenju PB i zaslužuje siru kliničku primenu.

## Introduction

PD, as a common neurodegenerative disease in clinics, has not yet been fully understood in its pathogenesis and mechanism, which may be related to drugs, society and patients' factors [1]. The incidence rate of this disease has shown a continuous rising trend, which has attracted great attention from the majority of medical staff and patients [2]. Patients will show non-motor symptoms, including sleep disorders, mental disorders, sensory disorders, autonomic nerve dysfunction, and motor symptoms, including myotonia, static tremor, bradykinesia, posture and gait abnormalities [3]. In addition to these well-documented clinical features, oxidative stress, mitochondrial dysfunction, and neuroinflammation are increasingly recognised as central contributors to PD progression. Emerging biomarkers such as glutare-doxin-1 (Grx1), peroxiredoxin-3 (Prx3), thioredoxin (Trx), and 8-hydroxy-2'-deoxyguanosine (8-OHdG) have been proposed to assess oxidative stress-mediated neurodegeneration, offering a deeper insight into disease pathophysiology. With the continuous development of the disease, it can lead to the death of patients, so active and effective treatment should be taken. At present, there is no cure for PD in the clinic; only drugs can be used to control the progression of the disease, and there is no safe and effective treatment [4]. The treatment of PD is mainly drug treatment, and the commonly used drug is Levodopa. It can treat PD by supplementing dopamine in neurons, but the effect of single drug treatment is limited, and increasing the dose will increase the adverse reactions of the drugs [5].

Additionally, long-term levodopa therapy is associated with motor fluctuations and dyskinesias, necessitating adjunct therapies that optimise its efficacy while minimising side effects. Pramipexole can significantly improve the clinical symptoms of patients with PD, enhance treatment efficacy, reduce the occurrence of adverse drug reactions, and improve the quality of life of patients [6]. However, the existing research is still relatively lacking [7]. As a dopamine D2/D3 receptor agonist, pramipexole not only complements Levodopa's effects but also exerts neuroprotective properties by modulating mitochondrial function, reducing oxidative damage, and possibly influencing neurosteroid levels such as dehydroepiandrosterone sulfate (DHEA-S). This study aims to evaluate whether pramipexole enhances Levodopa's therapeutic benefits by reducing oxidative stress through novel biomarkers and improving both motor and cognitive function in PD patients. Our study aims to explore the feasibility of pramipexole and Levodopa in the treatment of PD. By integrating novel oxidative stress and neurosteroid biomarkers, this research seeks to provide a more comprehensive understanding of the molecular mechanisms underlying PD progression and treatment response.

## Materials and methods

### General information

Ninety-two patients with PD who received income from our hospital from January 2022 to December 2024 were assigned to a single and combined group, with 46 patients each. The single group included 25 females and 21 males, with an average age of 67.52±2.43 years (49-86 years). The combined group included 24 females and 22 males, ranging from 49 to 87 years, with an average age of 67.89±5.31 years. No difference in basic characteristics between the two groups (P>0.05).

Inclusion criteria: (1) Diagnosis of idiopathic PD according to MDS clinical diagnostic criteria; (2) Hoehn & Yahr stage II-III; (3) PD duration between 2 and 10 years; (4) Good compliance and ability to actively follow medical advice; (5) Informed consent obtained from patients and their families. Exclusion criteria: (1) Intolerance to study drugs; (2) Severe hepatic or renal impairment; (3) History of major cerebrovascular events (e.g., cerebral haemorrhage or infarction); (4) Advanced PD (Hoehn & Yahr stage IV-V) or duration <2 years or >10 years.

### Methods

Single group: Patients were treated with Levodopa on the basis of routine treatment, with an initial dose of 125 mg/time, 3 times/day. After one week of administration, the dose is gradually increased according to the patient's condition and tolerance. The dose can be increased by 125 mg/time every 3-4 days, 3 times/day. After the patient's symptoms have improved and his condition is stable, maintain the drug dosage. The maximum dosage should not exceed 3000 mg/d.

Combined group: patients were treated with pramipexole based on the single group treatment (the usage and dosage of Levodopa were the same as those in the single group). The first dose was 0.125 mg/time, 3 times/day. After 1 week of treatment, the drug dose was increased according to the patient's condition and tolerance, with each increase of 0.125 mg/time, 3 times/day. The dosage of pramipexole can be increased by 0.125 mg/time, 3 times/day every 1 week. After the patient's condition improves and stabilises, the dosage of pramipexole can be maintained. The maximum dose of pramipexole should not exceed 4.5 mg/day. Both groups were treated continuously for 3 months.

### Biomarker evaluation

Serum levels of SOD, GSH, GSH-PX, and CAT were measured using commercially available ELISA kits (Nanjing Jiancheng Bioengineering Institute, Nanjing, China) according to manufacturer protocols. Grx1, Prx3, Trx, and 8-OHdG concentrations were quantified by sandwich ELISA (Cloud-Clone Corp., Wuhan, China), and all assays were validated with intra- and inter-assay CV<10%. DHEA-S levels were measured in serum using a chemiluminescent immunoassay (Beckman Coulter UniCel DxI 800, USA). Blood samples were collected in the morning after overnight fasting at baseline and after 3 months of therapy.

### Observation index

The treatment effects of the two groups were compared and evaluated according to the PDRating Scale (UPDRS). The obvious effect was that the score decreased by more than half after treatment; The effective rate is that the score decreases by one-fifth to one-half after treatment; The ineffectiveness is that the score decline after treatment does not meet the above standards or does not decline.

The improvement effect of two groups of patients after treatment. The UPDRS scores before and 6 weeks and 12 weeks after treatment were compared between the two groups.

QOL evaluation: QOL is the main result of evaluating the impact of drugs on PD patients. QOL was assessed with the PD quality of life rating scale, PDQ-39 [8]. PDQ-39 is a self-assessment scale with 39 questions, including 8 dimensions: exercise ability (10), daily activity behaviour (6), mental health (6), social support (3), physical discomfort (3), humiliation (4), cognition (4) and communication (3). The answers to each question of PDQ-39 have five options, with scores ranging from 0 to 4, and the highest possible score is 156. The higher the score, the lower the QOL of PD patients.

Intelligence and cognitive function: the cognitive function was assessed by the simple intelligence state examination scale (MMSE) and Montreal Cognitive Assessment Scale (MOCA). The MMSE scale includes 30 items in five aspects: orientation (10 points), immediate memory (3 points), attention and calculation (5 points), delayed recall (3 points) and language ability (9 points). Each item is expressed as correct (1 point) or wrong (0 points). The MOCA scale includes visual space and executive function (5 points), naming (3 points), attention (6 points), language ability (3 points), abstract ability (2 points), delayed recall (5 points) and orientation (6 points). Each item is scored as correct (1 point) or wrong (0 points).

Quality of life: the 39-item PD questionnaire (PDQ-39) was used to evaluate the quality of life, encompassing 8 aspects: physical activity, daily behaviour, mental health, disease humiliation, social support, cognition, communication, and physical discomfort. Each score was expressed by the frequency of items (0-4 points). The total score was 156. The higher the score, the worse the quality of life.

The adverse reactions of the two groups were compared, including nausea and vomiting, dizziness, sleepiness, hypotension, insomnia, epigastric discomfort, visual abnormalities, etc.

Oxidative Stress and Neuroprotection Analysis: Before and 3 months after treatment, the venous blood of the patient's elbow was taken on an empty stomach in the morning to detect the levels of SOD, GSH, GSH PX and CAT. Grx1, Prx3, Trx, and 8-OHdG were measured at baseline and after 3 months of therapy. DHEA-S levels were also assessed to evaluate potential neurosteroid-mediated neuroprotection.

### Statistical analysis

All research data were calculated and analysed by SPSS 20.0 statistical software. The measurement data between the two groups are expressed in (x̄ ± s) and a parallel t-test. The counting data are described in (%) and the parallel χ^2^ test. P<0.05 indicates that the difference is statistically significant.

## Results

### Comparison of therapy effects between the two groups

The total effective rate of the pramipexole-treated patients (93.48%) was significantly higher than that of the levodopa-treated patients (78.26%) (P<0.05) Table 1.

**Table 1:** Comparison of treatment effects between the two groups.

Groups	Obvious effective (n)	Effective (n)	Ineffective (n)	Total effective rate<br>[n (%)]	χ^2^ (df)	p-value
Single group<br>(n=46)	16	20	10	36 (78.26)	9.546 (1)	0.002
Combined group<br>(n=46)	31	12	3	43 (93.48)	-	-

### Comparison of UPDRS scores before and after treatment between the two groups

**Figure 1:**
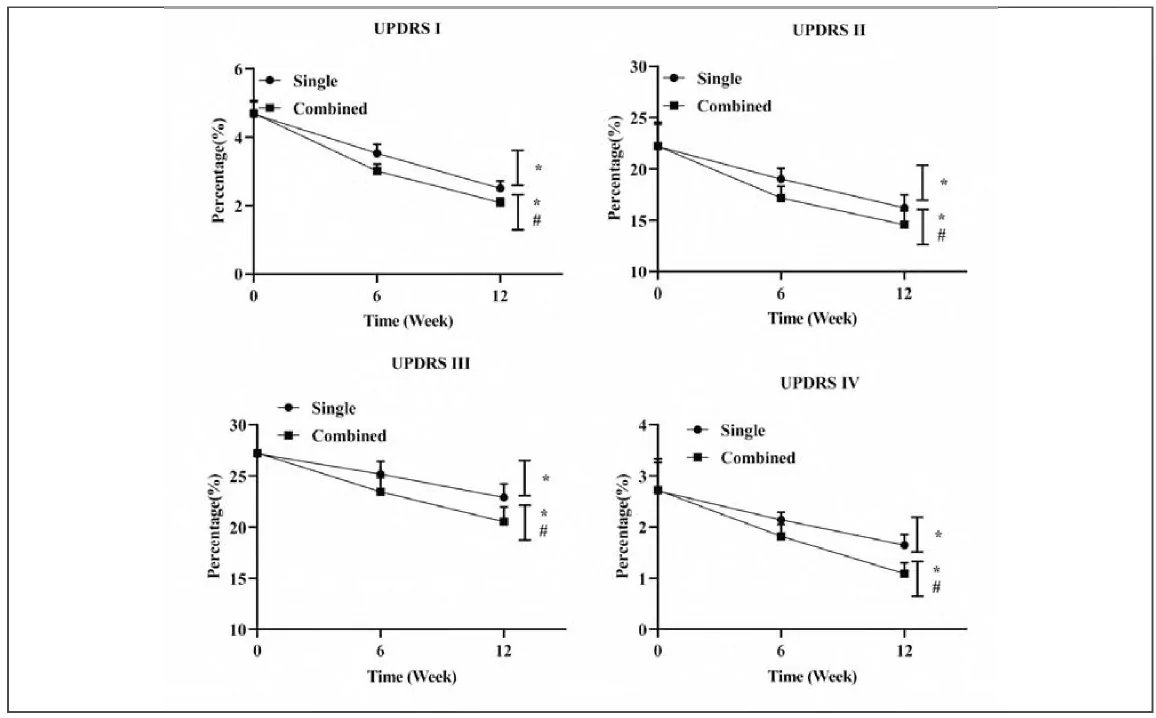
Comparison of UPDRS scores between the two groups before and after treatment.

The scores of UPDRS I-IV in the two groups before therapy were not significantly different (P>0.05). The scores of UPDRS I-III and UPDES IV in the combined group were substantially lower than those of levodopa-treated patients at 6 weeks and 12 weeks after therapy (P<0.05), indicating improved motor function and reduced disability, as shown in Figure 1.

### Comparison of adverse reactions between the two groups

The incidence of adverse drug reactions in the pramipexole-treated patients (6.52%) was markedly lower than that of levodopa-treated patients (23.91%) (P<0.05), suggesting a better safety profile for combination therapy, as shown in Table 2.

**Table 2:** Comparison of adverse reactions between the two groups

Groups	Nausea and<br>vomiting<br>(n)	Dizziness,<br>drowsiness and<br>lethargy (n)	Hypotension<br>(n)	Insomnia<br>(n)	Epigastric<br>discomfort<br>(n)	Visual<br>abnormality<br>(n)	Total occurrence of<br>adverse reactions<br>[n (%)]
Single group<br>(n=46)	3	2	1	1	2	2	11 (23.91)
Combined<br>group<br>(n=46)	1	1	0	0	0	1	3 (6.52)
χ^2^	-	-	-	-	-	-	11.721
F	-	-	-	-	-	-	<0.05

### Comparison of QOL evaluation results

**Figure 2:**
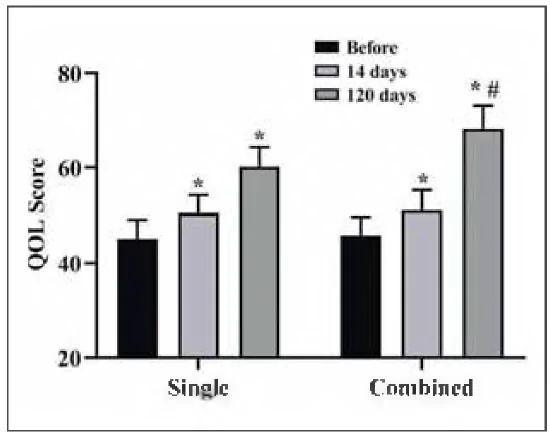
Comparison of the quality of life (QOL) between the two groups. PDQ-39 total scores at baseline, 14 days, and 3 months for both treatment groups. Higher scores indicate poorer QOL. Data are presented as mean ± SD. P < 0.05 vs. before treatment; # P < 0.05 vs. monotherapy group.

Before admission and after 14 days of intervention, no significant difference in QQL was found between the two groups (P>0.05). After 3 months of intervention, QOL in the pramipexole-treated patients was higher than in the levodopa-treated patients (P<0.05) (see Figure 2). The results showed that QOL of PD patients treated with pramipexole combined with Levodopa was significantly improved (P<0.05).

### Comparison of oxidative stress reaction between the two groups

**Figure 3:**
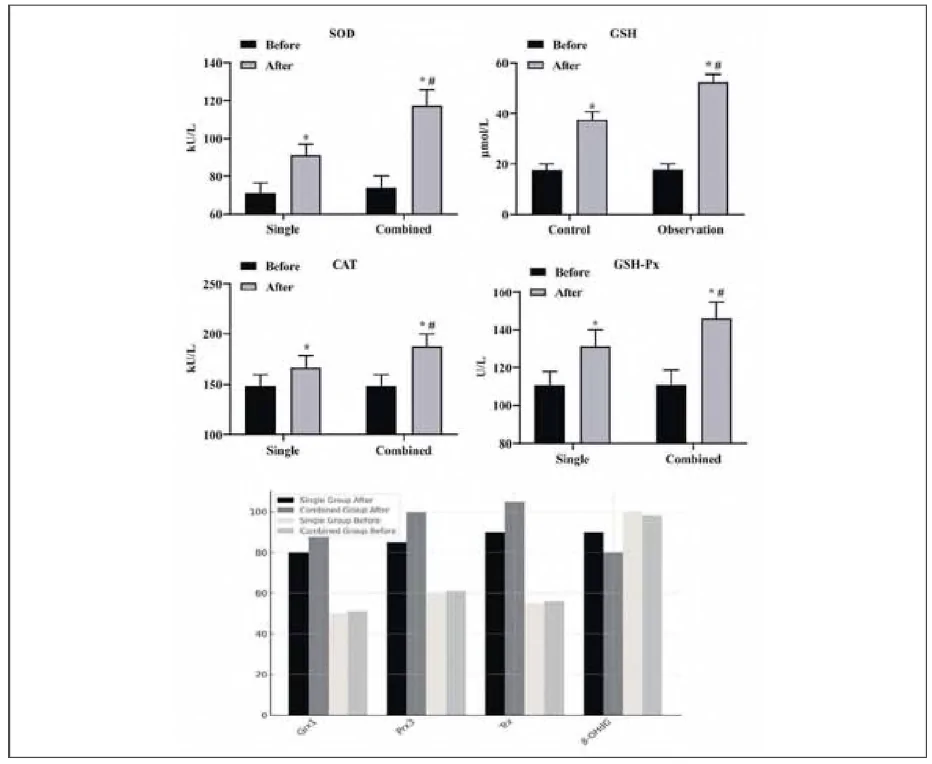
Changes in oxidative stress markers after 3 months of treatment. Serum levels of SOD, GSH, GSH-PX, CAT, Grx1, Prx3, Trx, and 8-OHdG in the levodopa monotherapy and pramipexole + levodopa groups. Data are expressed as mean ± SD. P < 0.05 vs. before treatment; # P < 0.05 vs. monotherapy group.

Before therapy, there was no significant difference in oxidative stress marker levels (P>0.05). After 3 months of therapy, the levels of SOD, GSH, GSH-PX, and CAT in patients were significantly higher than those before treatment (P<0.05), and levels in the pramipexole-treated patients were considerably higher than in the levodopa-treated patients (P<0.05), indicating reduced oxidative stress, as shown in Figure 3.

In addition to the traditional oxidative stress markers, novel biomarkers glutaredoxin-1 (Grx1), peroxiredoxin-3 (Prx3), thioredoxin (Trx), and 8-hydroxy-2'-deoxyguanosine (8-OHdG) were measured. Grx1, Prx3, and Trx levels increased significantly in the combination therapy group, suggesting enhanced redox balance. 8-OHdG, a marker of DNA oxidative damage, was significantly reduced in the combination group compared to the monotherapy group, indicating neuroprotective effects.

### Comparison of neurosteroid levels between the two groups

**Figure 4:**
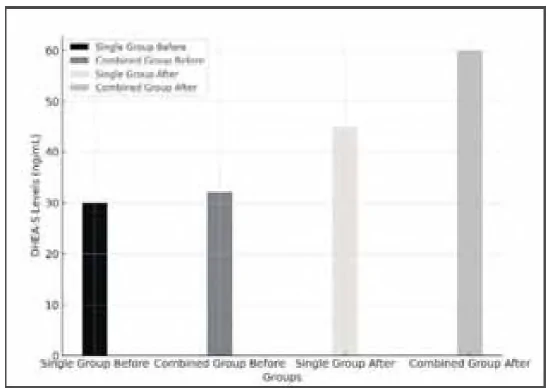
Comparison of neurosteroid (DHEA-S) levels between the two groups. Serum DHEA-S concentrations before and after 3 months of therapy. Data are shown as mean ± SD. P < 0.05 vs. before treatment; # P < 0.05 vs. monotherapy group.

Neurosteroid DHEA-S levels were measured to evaluate their potential role in cognitive and motor function improvements. Before treatment, there was no significant difference in DHEA-S levels between the two groups (P>0.05). After 3 months, DHEA-S levels were significantly higher in the pramipexole-treated group compared to the levodopa-only group (P<0.05). This increase correlated with higher MMSE and MoCA scores, suggesting a neurosteroid-mediated mechanism for cognitive enhancement (Figure 4). Before treatment, there was no significant difference in DHEA-S levels between the two groups (P>0.05). After 3 months, DHEA-S levels were significantly higher in the combination group compared to the monotherapy group (P<0.05). Pearson correlation analysis showed that post-treatment DHEA-S levels were positively correlated with MMSE scores (r=0.46, p<0.001) and MoCA scores (r=0.43, p=0.002), indicating that higher DHEA-S levels were associated with better cognitive performance.

### Comparison of the quality of life between the two groups

Before therapy, no significant difference in QOL scores was found between the two groups (P>0.05). After 3 months of treatment, the quality of life scores of patients were significantly higher than those before therapy (P<0.05). After 3 months of therapy, the quality of life scores of patients in pramipexole-treated patients were significantly higher than those in the levodopa-treated patients (P<0.05), as shown in Table 3.

**Table 3:** Comparison of quality of life between the two groups.

Groups	Single group (n=46)		Combined group (n=46)		χ^2^	P
	Pre-treatment	Post-therapy	Pre-treatment	Post-therapy	-	-
Physical activity	2.37±0.23	2.73±0.42	2.41±0.31	3.41±0.69	-	-
Daily behavior	2.16±0.25	2.94±0.36	2.12±0.28	3.56±0.72	-	-
Mental health	2.21±0.32	2.93±0.41	2.28±0.36	3.44±0.53	-	-
Disease humiliation	2.43±0.36	3.08±0.42	2.40±0.33	3.62±0.47	-	-
Social support	2.07±0.21	2.95±0.39	2.12±0.22	3.53±0.17	-	-
Cognition	2.31±0.23	2.86±0.78	2.26±0.30	3.68±0.72	-	-
Malaise	2.11±0.20	2.89±0.35	2.15±0.36	3.73±0.49	-	-
Communication	2.35±0.31	2.98±0.73	2.32±0.36	3.84±0.91	-	-
Total score	20.43±0.30	23.79±0.76	20.17±0.46	28.47±0.88	19.673	< 0.05

### Comparison of the intelligence MMSE and cognitive function MOCA scores between the two groups

**Figure 5:**
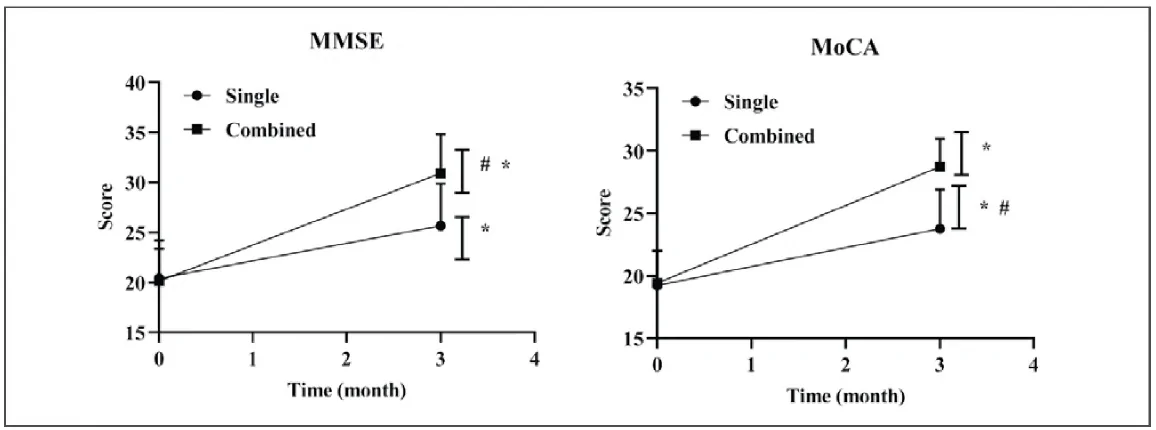
Comparison of cognitive function scores (MMSE and MoCA) between the two groups.MMSE and MoCA scores at baseline and after 3 months of treatment in both groups. Data are presented as mean ± SD. P < 0.05 vs. before treatment; # P < 0.05 vs. monotherapy group.

Before therapy, no significant difference in intelligence, MMSE and cognitive function MOCA scores was found between the two groups (P>0.05). After 3 months of therapy, the scores of intelligence MMSE and cognitive function MOCA of patients were significantly higher than those before therapy (P<0.05). After 3 months of therapy, the scores of intelligence MMSE and cognitive function MOCA of patients in the combined group were significantly higher than those in the single group (P<0.05), as shown in Figure 5.

## Discussion

Parkinson's disease is a common chronic disease of the extravertebral nervous system in elderly patients. The degenerative changes of dopamine neurons lead to the imbalance of dopamine and acetylcholine, and the decrease of dopamine content in the striatum. After the onset of the disease, the patient's condition will gradually worsen, which will not only affect their life and work, but also gradually lose their ability to take care of themselves. At present, PD has become a common disease threatening the quality of life of middle-aged and elderly people [8]. Drug therapy is generally used in clinics, but there is no specific drug that can cure the disease at present, and the progression of the disease can only be controlled through long-term drug use. However, long-term drug use will also have adverse effects on the body. Many patients will have different degrees of drug resistance after long-term drug use, which makes it impossible to control the progression of Parkinson's disease effectively. Emerging evidence suggests that oxidative stress and mitochondrial dysfunction play a crucial role in PD progression, leading to increased neuronal degeneration. The inclusion of oxidative stress biomarkers in this study, such as glutaredoxin-1 (Grx1), peroxiredoxin-3 (Prx3), thioredoxin (Trx), and 8-hydroxy-2'-deoxyguanosine (8-OHdG), allows for a deeper understanding of oxidative damage and its modulation by combination therapy. Therefore, effective drug treatment methods should be explored in the clinic [9]. Levodopa can supplement dopamine transmitters, so it can significantly improve the clinical symptoms of patients. However, with the increase in the dose of medopa, patients often experience fluctuations in symptoms, and adverse drug reactions will also increase [10]. In addition to motor complications, chronic levodopa use is linked to increased oxidative stress, which can exacerbate neurodegeneration. In this study, reductions in 8-OHdG and increases in Trx and Prx3 in the combination therapy group suggest enhanced neuroprotection and a potential antioxidative mechanism of pramipexole.

The total effective rate of the combined group was 93.48%, which was higher than that of the single group (78.26%) (P<0.05). There was no significant difference in the scores of UPDRS I, UPDRS II, UPDRS III and UPDRS IV between the two groups before treatment (P>0.05). The scores of UPDRS I, UPDRS II, UPDRS III and UPDRS IV in the combined group were significantly lower than those in the single group at 6 weeks and 12 weeks after treatment (P<0.05). The incidence of adverse drug reactions in the combined group was 6.52%, which was significantly lower than that in the single group (23.91%) (P<0.05). These findings are further supported by reductions in oxidative stress markers in the combination group, indicating that pramipexole may contribute to neuroprotection beyond its role as a dopamine receptor agonist.

In clinical practice, patients with Parkinson's disease (PD) are usually treated with pharmacological therapies. It is essential to provide effective and safe medications that can accelerate recovery and improve quality of life. Levodopa, typically administered in combination with a peripheral dopa-decarboxylase inhibitor, reduces dopamine metabolism in peripheral tissues. This increases the amount of Levodopa reaching the central nervous system, thereby enhancing its therapeutic effect [11]. However, levodopa monotherapy is often insufficient in addressing non-motor symptoms, such as cognitive decline. In this study, the inclusion of neurosteroid analysis - particularly dehydroepiandrosterone sulfate (DHEA-S) - enabled an investigation into how pramipexole may contribute to neurosteroid-mediated cognitive benefits. We found that DHEA-S levels were significantly higher in the combination therapy group, correlating with improved MMSE and MoCA scores, suggesting a potential role for pramipexole in cognitive protection. Levodopa remains effective in alleviating motor symptoms such as tremor and rigidity, improving muscle tone, and facilitating the recovery of motor function. It significantly reduces myotonia and bradykinesia, while also promoting the release of dopamine, norepinephrine, and other neurotransmitters. Nevertheless, the therapeutic effect of Levodopa is influenced by multiple factors, and its efficacy as monotherapy remains limited [12].

In addition, long-term use of Levodopa is associated with multiple adverse reactions [12]. Therefore, to ensure sustained efficacy, adjunctive therapies should be considered to reduce the required Levodopa dosage while maintaining drug safety [13]. In this study, the observed reduction in oxidative stress - reflected by increased Grx1, Prx3, and Trx - suggests that pramipexole may exert protective effects against oxidative damage, a key factor in PD progression.

Pramipexole was first marketed in Europe in 2009. Its gradual-release formulation allows for more precise delivery of the active ingredient while minimising the adverse effects of excessive dosing [14]. When used in combination with Levodopa, pramipexole provides superior therapeutic outcomes. As a nonergot dopamine D2/D3 receptor agonist, pramipexole differs markedly from traditional dopamine receptor agonists. Stimulating dopamine receptors in the striatum effectively alleviates clinical symptoms [15]. Beyond dopaminergic activity, pramipexole also exhibits antioxidant properties, modulates mitochondrial function, and increases neurosteroid levels, suggesting multiple mechanisms of neuroprotection.

Pramipexole demonstrates high affinity for dopamine receptors in the striatum and substantia nigra, influencing neuronal firing rates [16]. It protects dopaminergic cells from apoptosis induced by 1-methyl-4-phenylpyridinium (MPP ) and inhibits the production of quinone derivatives [17]. Furthermore, its antioxidant effects reduce cellular damage and death in the substantia nigra, thereby preserving dopaminergic neurons. Due to its high selectivity for the D2 receptor subfamily, pramipexole improves motor symptoms, protects neurons, delays the degeneration of substantia nigra cells, and is rapidly absorbed after oral administration, with relatively high bioavailability [18].

Clinical findings show that the rate of adverse reactions in the combination group was lower than in the Levodopa-only group. This suggests that combining Levodopa with pramipexole not only reduces adverse events but also prevents or delays motor complications. Combination therapy allows for reduced Levodopa dosing, extends the effective treatment window of L-DOPA, and provides direct dopaminergic stimulation at postsynaptic receptors, thereby enhancing dopamine release [19]. Overall, combined Levodopa-pramipexole therapy exerts synergistic effects, reduces Levodopa dosage requirements, improves drug safety, alleviates symptoms, and supports recovery in patients with PD [20][21].

### Analysis of research results

The primary outcome of this study was quality of life (QOL). After three months of treatment, QOL scores in the test group were significantly higher than those in the control group, particularly in domains such as motor ability, daily activities, mental health, and communication. As a secondary outcome, medication compliance in the test group was also significantly higher than in the control group after three months, encompassing adherence to dosing schedules, diet, and exercise.

On day 14, no significant differences in QOL or medication compliance were observed between the two groups. This was likely because all patients were hospitalised at that time, closely followed physician instructions, adhered to fixed medication schedules, maintained stable diets, and engaged in structured rehabilitation exercise. Thus, while both groups showed significant improvements compared with baseline, intergroup differences only became apparent after continued treatment. During hospitalisation, clinicians provided education to patients on PD and its pharmacological management, monitored drug interactions and adverse reactions, and offered ongoing consultation before and after discharge. These measures likely contributed to the overall improvement in medication adherence and self-management.

Biochemical markers also reflected significant therapeutic benefits. After three months of treatment, levels of SOD, GSH, GSH-Px, and CAT were significantly increased in both groups compared with baseline (P<0.05). Notably, the combined therapy group exhibited significantly higher levels of these markers than the levodopa-only group (P<0.05). In addition to these conventional oxidative stress markers, Grx1, Prx3, and Trx were significantly elevated in the combination group, while 8-OHdG was reduced. These findings suggest that pramipexole may enhance redox balance and provide additional neuroprotection.

Compared with Levodopa alone, combination therapy with pramipexole not only regulated oxidative stress responses but also improved mental health, motor function, and daily living activities, thereby slowing disease progression. Furthermore, the observed increase in DHEA-S levels in the combination group suggests that pramipexole may modulate neurosteroid pathways, potentially contributing to cognitive and emotional benefits.

### Study limitations

This study has several limitations. First, the absence of a placebo control group prevents definitive attribution of the observed benefits to combination therapy alone, as non-pharmacological factors may have influenced outcomes. Second, the relatively short follow-up period of three months limits the assessment of long-term effects on disease progression, neuroprotection, and cognitive outcomes. Third, the single-centre design may introduce selection bias related to patient demographics, referral patterns, and treatment practices, thereby limiting generalizability to broader PD populations. Finally, although the sample size was sufficient to detect short-term clinical differences, it may not have been large enough to capture less common adverse events or subtle biomarker changes. Longer follow-up studies are therefore needed to evaluate the sustained impact of pramipexole-levodopa combination therapy. Future multicenter, placebo-controlled trials with larger cohorts are warranted to validate and extend these findings.

In summary, pramipexole combined with Levodopa significantly improved clinical outcomes in patients with PD, with fewer adverse drug reactions compared with Levodopa monotherapy. The inclusion of novel biomarkers in this study highlights the potential of pramipexole to exert antioxidant and neurosteroid-mediated neuroprotective effects. These results support the clinical use and further exploration of pramipexole as part of an optimised therapeutic strategy for Parkinson's disease.

## Conclusion

Pramipexole combined with Levodopa has demonstrated significant efficacy in the treatment of Parkinson's disease (PD), improving motor symptoms, mental status, and quality of life while reducing the incidence of adverse reactions. Compared with Levodopa monotherapy, combination therapy produced superior outcomes not only in symptom relief but also in attenuating oxidative stress, as reflected by increased levels of antioxidant markers (SOD, GSH, GSH-Px, CAT) and novel redox regulators (Grx1, Prx3, Trx), along with reduced 8-OHdG levels. These findings suggest that pramipexole may exert neuroprotective effects by enhancing redox homeostasis and mitigating oxidative damage, a key contributor to PD progression.

In parallel, the observed increase in the neurosteroid dehydroepiandrosterone sulfate (DHEA-S) in the combination group correlated with improved cognitive function, indicating a potential role for pramipexole in neurosteroid-mediated neuroprotection. This cognitive benefit highlights the ability of pramipexole to address non-motor symptoms, such as cognitive decline, in addition to motor improvement. The concomitant enhancement of QOL and medication compliance further underscores the therapeutic value of combination therapy in optimising patient outcomes.

Collectively, these findings reinforce the potential of pramipexole to provide both symptomatic benefits and disease-modifying advantages that may slow PD progression. Future studies with larger cohorts and longer follow-up are warranted to determine whether the antioxidative and neurosteroid-modulating effects of pramipexole contribute to sustained neuroprotection and improved long-term prognosis in PD patients.

## Dodatak

### Conflict of interest statement

All the authors declare that they have no conflict of interest in this work.
